# Joint and Independent Associations of Gestational Diabetes and Depression With Childhood Obesity

**DOI:** 10.1001/jamanetworkopen.2025.59344

**Published:** 2026-02-18

**Authors:** Alicia K. Peterson, Lyndsay A. Avalos, Yeyi Zhu, Morgan Ashley Craft, Mara Greenberg, Amanda Ngo, Charles P. Quesenberry, Assiamira Ferrara

**Affiliations:** 1 Division of Research, Kaiser Permanente Northern California, Pleasanton; 2 Center for Upstream Prevention of Adiposity and Diabetes Mellitus (UPSTREAM), Division of Research, Kaiser Permanente Northern California, Pleasanton; 3 Bernard J. Tyson Kaiser Permanente School of Medicine, Pasadena, California; 4 Oakland Medical Center, Kaiser Permanente Northern California, Oakland; 5 Regional Perinatal Service Center, Kaiser Permanente Northern California, Oakland

## Abstract

**Importance:**

Childhood obesity has been independently associated with exposure to gestational diabetes and prenatal depression. Although these conditions frequently co-occur and may share biological pathways, their combined association with childhood obesity remains unknown.

**Objective:**

To examine whether exposure to prenatal depression and gestational diabetes is separately and jointly associated with childhood obesity.

**Design, Setting, and Participants:**

This prospective cohort study of births from 2011 to 2021 used data from Kaiser Permanente Northern California, an integrated health care system. Individuals receiving prenatal care were universally screened for depression and gestational diabetes and their children’s height and weight were monitored until age 10 years. Data analysis was performed from June 2024 to December 2025.

**Exposures:**

Gestational diabetes diagnosis and prenatal depression diagnosis and severity obtained from medical records.

**Main Outcomes and Measures:**

The primary outcome was childhood obesity, defined as Centers for Disease Control and Prevention body mass index *z *scores greater than or equal to the 95th percentile stratified by child age in years. Modified Poisson regression with robust SEs estimated risk ratios (RRs) for prenatal depression and gestational diabetes separately and jointly, adjusting for the birthing parent’s age, race and ethnicity, neighborhood deprivation, parity, and prenatal alcohol and tobacco use. Effect modification was assessed separately by statistical interactions and stratified models.

**Results:**

In this cohort study of 203 333 birthing parent–child pairs (mean [SD] age of birthing parents at the time of delivery, 30.8 [5.3] years; 104 214 male children [51.3%]), rates of childhood obesity increased with child’s age, from 14.6% (29 198 of 199 329 children) at age 2.0 to 4.9 years, to 16.5% (19 155 of 116 398 children) at age 5.0 to 7.9 years, and 21.8% (9798 of 44 894 children) at age 8.0 to 10.0 years. Prenatal depression was minimally associated with obesity (RR, 1.07 [95% CI, 1.04-1.10] for children aged 2.0-4.9 years; RR, 1.08 [95% CI, 1.04-1.12] for children aged 5.0-7.9 years; RR, 1.05 [95% CI, 1.00-1.11] for children aged 8.0-10.0 years). Gestational diabetes demonstrated larger effect estimates (RR, 1.29 [95% CI, 1.25-1.34] for children aged 2.0-4.9 years; RR, 1.45 [95% CI, 1.40-1.51] for children aged 5.0-7.9 years; RR, 1.39 [95% CI, 1.31-1.46] for children aged 8.0-10.0 years). Joint exposure to gestational diabetes and depression conferred the highest RRs compared with having neither exposure (RR, 1.33 [95% CI, 1.23-1.44] for children aged 2.0-4.9 years; RR, 1.54 [95% CI, 1.41-1.69] for children aged 5.0-7.9 years; RR, 1.43 [95% CI, 1.25-1.64] for children aged 8.0-10.0 years), with no evidence of interaction (*P* for interaction >0.10). After additionally adjusting for prepregnancy body mass index, joint results were attenuated, although the association for the 5.0 to 7.9 years age group remained especially robust.

**Conclusions and Relevance:**

In this cohort study, both prenatal depression and gestational diabetes were associated with childhood obesity risk, with larger effect sizes observed for gestational diabetes. Children exposed to both conditions had the greatest risk, although associations appeared additive rather than synergistic. These findings underscore the need for universal prenatal screening and risk stratification, along with targeted interventions for children exposed to these conditions.

## Key Points

**Question:**

Are gestational diabetes and prenatal depression independently or jointly associated with risk of childhood obesity in the first 10 years of life?

**Findings:**

In this cohort study of 203 333 birthing parent–child pairs from Northern California, both gestational diabetes and depression were independently significantly associated with increased risk of obesity, and children exposed to both conditions in utero had 33% significantly higher risk of obesity at age 2.0 to 4.9 years, 54% increased risk at age 5.0 to 7.9 years, and 43% increased risk at age 8.0 to 10.0 years, compared with those unexposed to either condition.

**Meaning:**

These findings underscore the need for universal prenatal screening and risk stratification, along with targeted interventions for children exposed to these conditions.

## Teaser

This cohort study examines whether prenatal depression and gestational diabetes are separately and jointly associated with childhood obesity.

## Introduction

US overweight and obesity rates have risen dramatically since 1990, particularly among children, with both prevalence and severity increasing with age.^1^ Projections indicate that by 2050, 1 in 3 children will have obesity.^1^ This growing epidemic poses substantial public health challenges, as obesity is associated with increased risk of serious conditions, such as diabetes, heart disease, hypertension, and certain cancers, ultimately reducing quality of life and placing heavy burdens on health care systems.^2^

Although diet and sedentary behavior are critical factors in obesity risk, emerging evidence highlights the important role of in-utero exposures in shaping long-term metabolic health, supporting the Developmental Origins of Health and Disease hypothesis.^3^ Gestational diabetes has been linked to metabolic changes in offspring, predisposing them to obesity and related cardiometabolic diseases later in life.^4^ Gestational diabetes is diagnosed in 7.8 per 100 US births, making it the most common metabolic complication in pregnancy, with sharp increases in recent years.^5^ Additionally, psychological disorders experienced during pregnancy are increasing, with the Centers for Disease Control and Prevention (CDC) indicating that depression diagnosed at delivery increased 7-fold between 2000 and 2015.^6^ Although prenatal depression has been associated with increased risk of gestational diabetes^7,8,9^ and altered fetal programming,^10^ the birthing parent’s chronic depression has also been shown be associated with an elevated risk of childhood obesity.^11^

Many previous studies examining gestational diabetes and depression independently have had insufficient racial diversity, reducing generalizability, and a lack of attention to how these exposures affect distinct stages of childhood growth and obesity risk. Moreover, the combined associations of prenatal depression and gestational diabetes with childhood obesity remain unexamined despite shared biological pathways. Prenatal depression and gestational diabetes may both induce a proinflammatory state, which, in turn, increases the risk of childhood obesity in the offspring.^12,13^ Prenatal depression heightens hypothalamic-pituitary-adrenal axis activity and increases the birthing parent’s cortisol exposure,^14^ which can cross the placenta and has been associated with poor birth outcomes and accelerated infant adiposity gain.^15^ These findings support a role for glucocorticoid-mediated fetal programming in shaping later childhood obesity risk.^16,17^ Pregnancy hyperglycemia contributes to oxidative stress, inflammation, and elevated cord C-peptide levels, promoting fetal adiposity.^18,19^ Further supporting possible shared or additive pathways, a recent systematic review found that prenatal depression was associated with increased gestational diabetes risk, and that comorbidity is associated with worse pregnancy outcomes than either condition alone.^20^

To further elucidate the possible association of birthing parent’s depression and gestational diabetes with the risk of childhood obesity in the offspring, we aimed to evaluate whether in-utero exposure to prenatal depression and gestational diabetes was associated with childhood obesity risk over 10 years of follow-up within an integrated health care system with universal gestational diabetes and prenatal depression screening. In addition, we sought to determine whether co-occurrence of both conditions had a synergistic effect—defined here as a joint effect greater than expected according to individual effects—on obesity risk across different childhood stages. We hypothesized that children exposed in utero to both prenatal depression and gestational diabetes would have increased risk of obesity during childhood, with potentially greater early childhood associations when developmental processes are most sensitive to prenatal influences.

## Methods

### Study Setting

This population-based prospective cohort study took place within Kaiser Permanente Northern California (KPNC), an integrated health care system serving approximately 4.6 million members (approximately 60 000 pregnancies annually) and covering 32.5% of the Northern California population, with demographics reflective of the geographic area (>30 000 square miles).^21^ Study data were obtained from electronic health records (EHRs) and administrative databases, including information on diagnoses, visits, prescriptions, laboratory results, and census-level characteristics. As part of standard prenatal care, pregnant individuals are screened for depression at multiple time points^22^ and universally for gestational diabetes. This study was approved by the institutional review board and human participants committee of the Kaiser Foundation Research Institute, with an exemption given for written consent. Methods and reporting for this cohort study adhered to the Strengthening the Reporting of Observational Studies in Epidemiology (STROBE) reporting guidelines.

### Analytic Cohort

We included all adult pregnancies (ages 18-54 years) occurring at KPNC from 2011 to 2021, selecting 2011 because prepregnancy body mass index (BMI; calculated as weight in kilograms divided by height in meters squared) became readily available within the EHR and 2021 to ensure children would be at least aged 2 years at the start of the funding period (January 2024), making them compatible to compute BMI *z* scores using CDC growth charts.^23^ Of the 371 124 individuals with a live birth during this period, we excluded 90 380 parent-child pairs due to missing child height and/or weight measurements taken within 30 days of each other for children younger than 5.0 years or within 60 days for children aged 5.0 to 9.9 years. We further excluded 14 725 birthing parents without a recorded gestational diabetes pregnancy screening, 300 with prepregnancy diabetes, and 862 with a malignant cancer diagnosis. To ensure independence of each observation, we included only singleton pregnancies, removing 4779 multiple gestations. Additionally, to maintain model independence, we retained only the birthing parent’s eldest child available in the EHR, removing 56 745 younger siblings. Each child contributed 1 observation per age group and could appear in multiple age-specific analyses; because each model was estimated separately, analyses remained statistically independent. As anticipated, the number of eligible children decreased in older age groups due to not yet aging into the category or no longer receiving KPNC care (eFigure 1 in Supplement 1). Those removed for missing data were demographically similar to those included in the analysis (eTable 1 in Supplement 1).

### Prenatal Depression and Severity

Prenatal depression was defined as having 1 or more *International Classification of Diseases, Ninth Revision* and *International Statistical Classification of Diseases and Related Health Problems, Tenth Revision* code documented within the EHR from the first day of the last menstrual period through the day before delivery (eAppendix in Supplement 1).^22^ To assess how depression treatment (medication or psychotherapy) during pregnancy influenced the risk of childhood obesity, we extracted EHR prescriptions for antidepressants (eAppendix in Supplement 1)^24^ in addition to psychotherapy appointments, defined as any psychotherapy visit 30 minutes or longer during pregnancy.

In addition, for depression symptom severity, we utilized the validated 9-item Patient Health Questionnaire (PHQ-9)^25^ universally administered during prenatal KPNC visits since 2013. We only used PHQ-9 data available from 2013 onward and highest recorded PHQ-9 during pregnancy for each individual; missing data were not imputed. The individual’s highest PHQ-9 score (range, 0-27) during pregnancy was categorized^22,24,26^ as none (0-4), mild (5-9), moderate (10-14), and severe (≥15) to examine potential dose-response relationships. Some patients exhibit high depressive symptoms without clinical diagnosis.^27^

### Gestational Diabetes

KPNC universally screens for gestational diabetes in pregnancy (>96% of pregnancies) at approximately 24 to 28 weeks’ gestation using a 50-g, 1-hour glucose challenge. If initial screening results are abnormal (≥140.5 mg/dL; to convert to millimoles per liter, multiply by 0.0555), members undergo a 100-g, 3-hour oral glucose tolerance test (OGTT) following a 12-hour fast. Gestational diabetes cases were identified and recorded in the EHR either by meeting (1) 2 or more plasma glucose values from the OGTT meeting Carpenter-Coustan thresholds recommended by the American College of Obstetricians and Gynecologists,^28^ or (2) fasting glucose levels greater than or equal to 91.9 mg/dL, either alone or during the OGTT, following a screening value greater than or equal to 180 mg/dL.

### Childhood Obesity

Childhood obesity was determined using height and weight measurements taken at well-child visits and categorized into childhood life stages^29,30^ depending on age in years at time of measurement: 2.0 to 4.9, 5.0 to 7.9, and 8.0 to 10.0 years. EHR height and weight measurements were used to determine BMI. Age-specific and sex-specific BMI *z *scores were calculated to classify obesity (≥95th percentile) using CDC growth references, valid for ages 2 to 20 years.^23^ Biologically implausible values, defined by CDC modified BMI *z* scores less than −4 or greater than 8, were removed. After data cleaning, a child was considered to have the outcome for a given life stage if obesity was identified at any point within that period, based on highest BMI *z* score recorded for each child. Longitudinal continuous BMI was not utilized due to nonlinear growth patterns across ages 2 to 10 years (eFigure 2 in Supplement 1).

### Covariates

Covariates were chosen a priori on the basis of conceptual frameworks and prior literature identifying factors associated with both birthing parent metabolic and mental health conditions and childhood obesity. These included individual-level and neighborhood-level characteristics abstracted from the EHR: birthing parent’s age at delivery (<25, 25-29, 30-34, or 35-54 years), birthing parent’s self-identified race and/or ethnicity (American Indian or Alaska Native, Asian or Pacific Islander, Black, Hispanic, White, multiracial, other [ie, any race or ethnicity not otherwise specified], or unknown), Neighborhood Deprivation Index quartile (assigned according to census tract corresponding to longest residential address from 2 years prior to last menstrual period through 20 weeks’ gestation), parity (first-born, second-born, and third-born or higher), alcohol use during pregnancy (yes or no), and smoking during pregnancy (yes or no). Models did not adjust for child sex and age at assessment as these were accounted for in the outcome.^23^ Covariates with missing data (<3%) were imputed using multiple imputation.

### Statistical Analysis

Data analysis was performed from June 2024 to December 2025. For each of the 3 life-stage analyses, we calculated prevalence of prenatal depression, gestational diabetes, and childhood obesity, as well as distributions of each covariate. Multivariable modified Poisson regression with robust SEs was used for point and interval estimation of risk ratios (RRs) owing to the outcome of childhood obesity not being rare. We first ran separate regression models to examine associations of prenatal depression with childhood obesity and of gestational diabetes with childhood obesity. Prenatal depression was individually assessed by separate models in 3 ways: diagnosed depression (yes or no), diagnosed depression with or without treatment reference to no depression, and depression symptom severity based on PHQ-9 score category. Subsequently, within each life stage, we examined the joint effect of exposures on obesity risk by defining a 4-level dummy variable: (1) no prenatal depression and no gestational diabetes (reference), (2) gestational diabetes only, (3) prenatal depression only, and (4) both prenatal depression and gestational diabetes.

To further evaluate potential effect modification by co-occurring prenatal depression and gestational diabetes on childhood obesity, we added an interaction term (prenatal depression [yes or no] × gestational diabetes [yes or no]) in nonjoint models. *P* < .10 for this term was considered evidence of statistical interaction. We also stratified models by prenatal depression diagnosis (yes or no) and PHQ-9 severity (0-4, 5-9, 10-14, and ≥15) to examine whether the association between gestational diabetes and childhood obesity varied by depression.

As a secondary analysis, we considered the role of prepregnancy BMI in all models. Higher prepregnancy BMI is associated with increased likelihood of excessive gestational weight gain and elevated gestational adiposity,^31^ which contribute to insulin resistance and development of gestational diabetes, as well as metabolic and inflammatory changes that may increase prenatal depression risk.^32,33^ Additionally, the birthing parent’s prepregnancy BMI is a factor associated with childhood obesity, independently of gestational diabetes and prenatal depression.^34^ Because prepregnancy BMI is correlated with gestational weight gain, which may lie on the causal pathway between these exposures and childhood obesity, adjusting for it in the primary analysis could introduce bias by attenuating estimated associations of gestational diabetes and prenatal depression. However, given its association with both exposures and outcome, we conducted a secondary analysis adjusting for prepregnancy BMI to assess robustness of our findings. Prepregnancy BMI was categorized as underweight (<18.5), normal weight (≥18.5 and <25.0), overweight (≥25.0 and <30.0), and obese (≥30.0).

All models were adjusted for predetermined covariates; a 2-sided α level of .05 was used for statistical significance. Given the very large sample sizes under study, model goodness-of-fit testing is of minimal use given the tendency to reject the null for minor model deviations of no practical consequence (as is the case for any large sample statistical testing). Alternatively, for each regression model, we calculated the components of the Hosmer-Lemeshow test (with no significance testing) consisting of observed and expected number of events within each decile of estimated values for the sample used to fit the model. In general, differences between observed and expected events across deciles groups for all regression models were reasonably small, indicating no serious deviations from model assumption that would result in misleading point and interval estimates. Analyses were conducted using SAS statistical software version 3.81 (SAS Institute) for data cleaning and R statistical software version 4.3.1 (R Project for Statistical Computing) for modeling.

## Results

The final analytic sample included 203 333 unique birthing parent–child pairs, with sample sizes for obesity analyses within age groups in years as follows: 199 329 children aged 2.0 to 4.9 years, 116 398 children aged 5.0 to 7.9 years, and 44 894 children aged 8.0 to 10.0 years. In the overall analytic sample (including each parent-child pair only once across all age groups), the mean (SD) age of birthing parents was 30.8 (5.3) years at the time of delivery. The racial and ethnic distribution was 0.4% American Indian or Alaska Native (733 individuals), 27.4% Asian or Pacific Islander (55 797 individuals), 6.4% Black (13 012 individuals), 25.7% Hispanic (52 322 individuals), 35.7% White (72 655 individuals), 3.0% multiracial or other (6130 individuals), and 1.3% missing or unknown (2684 individuals). Average prepregnancy BMI was equivalent to having overweight (mean [SD], 26.3 [5.3]). Only 22 708 birthing parents (11.2%) reported alcohol use during pregnancy, and 4800 (2.4%) smoked during pregnancy. Slightly more male children were represented in the sample (104 214 boys [51.3%]). Birthing parent demographics by child age category were similar (Table 1).

**Table 1.** Demographic and Clinical Characteristics of Birthing Parent–Child Pairs by Age Group Analysis

Characteristic	Birthing parent–child pairs, No. (%) (N = 203 333)		
	Child age 2.0-4.9 y (n = 199 329)	Child age 5.0-7.9 y (n = 116 398)	Child age 8.0-10.0 y (n = 44 894)
Birthing parents			
Age, mean (SD), y	30.8 (5.3)	30.9 (5.3)	31.0 (5.3)
<25	24 756 (12.4)	14 545 (12.5)	5338 (11.9)
25-29	52 477 (26.3)	30 566 (26.3)	11 698 (26.1)
30-34	73 107 (36.7)	42 377 (36.4)	16 278 (36.3)
35-54	48 989 (24.6)	28 910 (24.8)	11 580 (25.8)
Race and ethnicity			
American Indian or Alaska Native	716 (0.4)	393 (0.3)	159 (0.4)
Asian or Pacific Islander	54 904 (27.5)	31 697 (27.2)	12 182 (27.1)
Black	12 713 (6.4)	7359 (6.3)	2775 (6.2)
Hispanic	51 122 (25.6)	30 118 (25.9)	11 607 (25.9)
White	71 225 (35.7)	41 831 (35.9)	16 160 (36.0)
Multiracial or any other race not otherwise specified	6000 (3.0)	3814 (3.3)	1678 (3.7)
Unknown or missing	2649 (1.3)	1186 (1.0)	333 (0.7)
Neighborhood Deprivation Index, quartile			
First (least deprived)	32 495 (16.3)	17 562 (15.1)	6734 (15.0)
Second	61 259 (30.7)	36 295 (31.2)	14 357 (32.0)
Third	58 379 (29.3)	34 886 (30.0)	13 574 (30.2)
Fourth (most deprived)	47 078 (23.6)	27 582 (23.7)	10 201 (22.7)
Missing	118 (0.1)	73 (0.1)	28 (0.1)
Prepregnancy BMI, mean (SD)^a^	26.3 (6.1)	26.2 (6.1)	26.2 (6.0)
<18.5	16 091 (8.1)	9074 (7.8)	3097 (6.9)
≥18.5 and <25.0	77 159 (38.7)	46 149 (39.6)	18 160 (40.5)
≥25.0 and <30.0	54 486 (27.3)	31 717 (27.2)	12 526 (27.9)
≥30.0	45 526 (22.8)	26 051 (22.4)	9953 (22.2)
Missing	6067 (3.0)	3407 (2.9)	1158 (2.6)
Parity			
0	111 118 (55.7)	59 993 (51.5)	20 352 (45.3)
1	55 666 (27.9)	35 389 (30.4)	15 937 (35.5)
≥2	32 485 (16.3)	20 980 (18.0)	8588 (19.1)
Missing	60 (<0.1)	36 (<0.1)	17 (<0.1)
Smoking, during pregnancy			
No	194 673 (97.7)	113 471 (97.5)	43 796 (97.6)
Yes	4630 (2.3)	2904 (2.5)	1083 (2.4)
Missing	26 (<0.1)	23 (<0.1)	15 (<0.1)
Alcohol, during pregnancy			
No	176 484 (88.5)	103 314 (88.8)	39 712 (88.5)
Yes	22 283 (11.2)	12 850 (11.0)	5090 (11.3)
Missing	562 (0.3)	234 (0.2)	92 (0.2)
Prenatal depression diagnosis			
No	164 107 (82.3)	96 730 (83.1)	37 517 (83.6)
Yes	35 222 (17.7)	19 668 (16.9)	7377 (16.4)
Depression severity score^b^			
0-4	110 244 (55.3)	63 985 (55.0)	23 869 (53.2)
5-9	45 168 (22.6)	25 378 (21.8)	8694 (19.4)
10-14	13 218 (6.6)	7328 (6.3)	2511 (5.6)
≥15	6248 (3.1)	3436 (3.0)	1148 (2.5)
Missing	24 451 (12.3)	16 271 (13.9)	8672 (19.3)
Gestational diabetes diagnosis			
No	181 152 (90.9)	106 201 (91.2)	41 202 (91.8)
Yes	18 177 (9.1)	10 197 (8.8)	3692 (8.2)
Children			
Sex assigned at birth			
Girls	97 120 (48.7)	56 656 (48.7)	21 864 (48.7)
Boys	102 209 (51.3)	59 742 (51.3)	23 030 (51.3)
No. of BMI measurements, mean (SD)	6.2 (5.6)	8.7 (5.9)	11.4 (7.0)
Obesity, age 2.0-4.9 y^c^			
No	170 131 (85.4)	NA	NA
Yes	29 198 (14.6)	NA	NA
Obesity, age 5.0-7.9 y^c^			
No	NA	97 243 (83.5)	NA
Yes	NA	19 155 (16.5)	NA
Obesity, age 8.0-10.0 y^c^			
No	NA	NA	35 096 (78.2)
Yes	NA	NA	9798 (21.8)

Abbreviations: BMI, body mass index; NA, not applicable.

^a^ BMI is calculated as weight in kilograms divided by height in meters squared.

^b^ Depression severity was determined by the 9-item Patient Health Questionnaire. Universal perinatal depression screening was implemented as part of standard prenatal care at Kaiser Permanente Northern California beginning in 2013.

^c^ Obesity is defined by a sex-specific and age-specific body mass index *z* score greater than or equal to the 95th percentile.

The prevalence of prenatal depression in birthing parents was 17.7% (35 222 birthing parents) among children aged 2.0 to 4.9 years (birth years 2011-2020), 16.9% (19 668 birthing parents) among those aged 5.0 to 7.9 years (birth years 2011-2017), and 16.4% (7377 birthing parents) among those aged 8.0 to 10.0 years (birth years 2011-2014). The prevalence of gestational diabetes in birthing parents was 9.1% (18 177 birthing parents) among children aged 2.0 to 4.9 years, 8.8% (10 197 birthing parents) among those aged 5.0 to 7.9 years, and 8.2% (3692 birthing parents) among those aged 8.0 to 10.0 years. Obesity prevalence increased with age, affecting 14.6% of children (29 198 children) aged 2.0 to 4.9 years, 16.5% of children (19 155 children) aged 5.0 to 7.9 years, and 21.8% of children (9798 children) aged 8.0 to 10.0 years (Table 1).

There was a small increase in childhood obesity risk associated with birthing parent prenatal depression (RR, 1.07 [95% CI, 1.04-1.10] for children aged 2.0-4.9 years; RR, 1.08 [95% CI, 1.04-1.12] for children aged 5.0-7.9 years; RR, 1.05 [95% CI, 1.00-1.11] for children aged 8.0-10.0 years). A similar pattern was observed for untreated depression compared with no depression (RR, 1.07 [95% CI, 1.03-1.10] for children aged 2.0-4.9 years; RR, 1.09 [95% CI, 1.04-1.14] for children aged 5.0-7.9 years; RR, 1.05 [95% CI, 0.98-1.11] for children aged 8.0-10.0 years). No clear pattern was observed between depression symptom severity and childhood obesity risk across different age groups. Results were attenuated but similar in secondary analyses when additionally adjusting for prepregnancy BMI (Table 2).

**Table 2.** Associations of Prenatal Depression, Depression Treatment Status, and Depression Severity With Childhood Obesity Across Age Groups^a^

Variable	Age 2.0-4.9 y			Age 5.0-7.9 y			Age 8.0-10.00 y		
	No. of children	RR (95% CI)		No. of children	RR (95% CI)		No. of children	RR (95% CI)	
		Main model^b^	Main model and prepregnancy BMI^c^		Main model^b^	Main model and prepregnancy BMI^c^		Main model^b^	Main model and prepregnancy BMI^c^
Prenatal depression diagnosis^d^									
No	170 256	1 [Reference]	1 [Reference]	100 729	1 [Reference]	1 [Reference]	39 394	1 [Reference]	1 [Reference]
Yes	29 073	1.07 (1.04-1.10)	1.02 (0.99-1.05)	15 669	1.08 (1.04-1.12)	1.01 (0.98-1.05)	5500	1.05 (1.00-1.11)	0.99 (0.95-1.04)
Prenatal depression diagnosis and treatment status^e^									
No depression	170 256	1 [Reference]	1 [Reference]	100 729	1 [Reference]	1 [Reference]	39 394	1 [Reference]	1 [Reference]
Depression with treatment	11 058	1.07 (1.02-1.12)	1.00 (0.96-1.05)	5891	1.06 (1.00-1.12)	0.98 (0.93-1.04)	2153	1.06 (0.98-1.15)	0.99 (0.92-1.07)
Depression without treatment	18 015	1.07 (1.03-1.10)	1.02 (0.99-1.06)	9778	1.09 (1.04-1.14)	1.03 (0.99-1.08)	3347	1.05 (0.98-1.11)	0.99 (0.94-1.05)
Prenatal depression symptom severity^f^									
0-4	73 929	1 [Reference]	1 [Reference]	31 797	1 [Reference]	1 [Reference]	2114	1 [Reference]	1 [Reference]
5-9	31 900	1.05 (1.02-1.09)	1.04 (1.00-1.07)	13 673	1.10 (1.05-1.14)	1.07 (1.03-1.12)	896	1.24 (1.07-1.45)	1.21 (1.04-1.40)
10-14	9432	1.09 (1.03-1.14)	1.07 (1.02-1.12)	4003	1.06 (0.99-1.13)	1.03 (0.96-1.10)	279	0.83 (0.62-1.09)	0.83 (0.62-1.09)
≥15	4542	1.19 (1.11-1.26)	1.14 (1.07-1.21)	1950	1.10 (1.00-1.20)	1.05 (0.96-1.14)	135	1.16 (0.84-1.59)	1.12 (0.82-1.53)

Abbreviations: BMI, body mass index; RR, risk ratio.

^a^ Childhood obesity is defined by a sex-specific and age-specific BMI (calculated as weight in kilograms divided by height in meters squared) *z* score greater than or equal to 95th percentile.

^b^ The main model is adjusted for the birthing parent’s age at time of delivery, race and ethnicity, Neighborhood Deprivation Index, parity, alcohol use in pregnancy, smoking in pregnancy.

^c^ The main model plus prepregnancy BMI is the main model additionally adjusted for prepregnancy BMI.

^d^ Depression exposures are assessed by separate regression models.

^e^ Treatment is defined by either psychiatry or medications.

^f^ Depression severity was determined by the 9-item Patient Health Questionnaire. Universal perinatal depression screening was implemented as part of standard prenatal care at Kaiser Permanente Northern California beginning in 2013 reflecting a slightly smaller sample size.

Gestational diabetes exposure was associated with increased risk of obesity at all stages (RR, 1.29 [95% CI, 1.25-1.34] for children aged 2.0-4.9 years; RR, 1.45 [95% CI, 1.40-1.51] for children aged 5.0-7.9 years; RR, 1.39 [95% CI, 1.31-1.46] for children aged 8.0-10.0 years). Results remained significant after adjusting for prepregnancy BMI (Table 3).

**Table 3.** In-Utero Exposure to Gestational Diabetes in Association With Childhood Obesity Risk Across Age Groups^a^

Variable	Age 2.0-4.9 y			Age 5.0-7.9 y			Age 8.0-10.0 y		
	No. of children	RR (95% CI)		No. of children	RR (95% CI)		No. of children	RR (95% CI)	
		Main model^b^	Main model and prepregnancy BMI^c^		Main model^b^	Main model and prepregnancy BMI^c^		Main model^b^	Main model and prepregnancy BMI^c^
No gestational diabetes	181 152	1 [Reference]	1 [Reference]	106 201	1 [Reference]	1 [Reference]	41 202	1 [Reference]	1 [Reference]
Gestational diabetes	18 177	1.29 (1.25-1.34)	1.09 (1.05-1.12)	10 197	1.45 (1.40-1.51)	1.17 (1.13-1.22)	3692	1.39 (1.31-1.46)	1.12 (1.06-1.18)

Abbreviations: BMI, body mass index; RR, risk ratio.

^a^ Obesity is defined by a sex-specific and age-specific BMI (calculated as weight in kilograms divided by height in meters squared) *z* score greater than or equal to the 95th percentile.

^b^ Main model is adjusted for the birthing parent’s age at time of delivery, race and ethnicity, Neighborhood Deprivation Index, parity, alcohol use in pregnancy, smoking in pregnancy.

^c^ Main model plus prepregnancy BMI is main model additionally adjusted for prepregnancy BMI.

When examining joint associations of exposure to both prenatal depression and gestational diabetes, there was evidence of increased childhood obesity risk compared with having neither condition (RR, 1.33 [95% CI, 1.23-1.44] for children aged 2.0-4.9 years; RR, 1.54 [95% CI, 1.41-1.69] for children aged 5.0-7.9 years; RR, 1.43 [95% CI, 1.25-1.64] for children aged 8.0-10.0 years) (Table 4). Results were attenuated after additionally adjusting for prepregnancy BMI in secondary analyses; however, results remained particularly robust for ages 5.0 to 7.9 years (Table 4). No effect modification was observed when testing the statistical interaction between gestational diabetes and prenatal depression exposure (either diagnosis or severity) on childhood obesity risk at any life stage (eTable 2 in Supplement 1).

**Table 4.** Joint Effects of In-Utero Exposure to Both Gestational Diabetes and Prenatal Depression in Association With Childhood Obesity Risk^a^

Variable	Age 2.0-4.9 y			Age 5.0-7.9 y			Age 8.0-10.0 y		
	No. of children	RR (95% CI)		No. of children	RR (95% CI)		No. of children	RR (95% CI)	
		Main model^b^	Main model and prepregnancy BMI^c^		Main model^b^	Main model and prepregnancy BMI^c^		Main model^b^	Main model and prepregnancy BMI^c^
No gestational diabetes and no depression	154 580	1 [Reference]	1 [Reference]	91 788	1 [Reference]	1 [Reference]	36 119	1 [Reference]	1 [Reference]
Depression only	26 572	1.07 (1.04-1.10)	1.02 (0.99-1.05)	14 413	1.08 (1.04-1.12)	1.02 (0.98-1.06)	5083	1.05 (1.00-1.11)	0.99 (0.94-1.05)
Gestational diabetes only	15 676	1.30 (1.25-1.35)	1.09 (1.06-1.13)	8941	1.46 (1.40-1.52)	1.18 (1.13-1.23)	3275	1.39 (1.31-1.47)	1.12 (1.06-1.19)
Gestational diabetes and depression	2501	1.33 (1.23-1.44)	1.06 (0.98-1.15)	1256	1.54 (1.41-1.69)	1.17 (1.06-1.28)	417	1.43 (1.25-1.64)	1.10 (0.96-1.25)

Abbreviations: BMI, body mass index; RR, risk ratio.

^a^ Obesity is defined by a sex-specific and age-specific BMI (calculated as weight in kilograms divided by height in meters squared) *z* score greater than or equal to the 95th percentile.

^b^ Main model is adjusted for the birthing parent’s age at time of delivery, race and ethnicity, Neighborhood Deprivation Index, parity, alcohol use in pregnancy, smoking in pregnancy.

^c^ Main model plus prepregnancy BMI is main model additionally adjusted for prepregnancy BMI.

## Discussion

This population-based cohort study found that prenatal depression and gestational diabetes were independently associated with increased obesity risk in offspring. Prenatal depression was minimally associated with risk of childhood obesity (5%-8% increased risk across offspring’s age groups). Gestational diabetes was associated with a higher risk of childhood obesity (29%-45% increased risk across offspring’s age groups). Children exposed to both conditions in utero had 33% higher risk of obesity at ages 2.0 to 4.9, 54% higher risk at ages 5.0 to 7.9 years, and 43% higher risk at ages 8.0 to 10.0 years compared with those unexposed to either condition. Adjustment for birthing parent’s prepregnancy BMI attenuated these joint associations, but they remained robust for obesity risk in children aged 5.0 to 7.9 years.

To our knowledge, no prior study has investigated the joint association of in-utero exposure to both prenatal depression and gestational diabetes with childhood obesity. Prenatal depression and gestational diabetes may independently and synergistically promote a proinflammatory intrauterine environment that increased susceptibility to later obesity.^12,13^ The placenta regulates fetal exposure to the birthing parent’s hormones, including cortisol, which is often elevated in depression. Normally, placental 11β-hydroxysteroid dehydrogenase type 2 converts cortisol to cortisone, but reduced enzyme activity in depression may increase cortisol exposure and later adiposity.^35^ Depression during pregnancy may also contribute to rapid infant weight gain and parenting practices that influence childhood health behaviors.^11,36^ Prenatal depression has been associated with higher gestational diabetes risk,^7,8,9^ and gestational diabetes has been associated with prenatal depression,^37^ indicating potential bidirectionality. Gestational diabetes–related hyperglycemia leads to fetal hyperinsulinemia and increased fat cell formation via insulin-driven adipogenesis, placental lipolysis, and transfer of free fatty acids.^38^ Depression and obesity in the birthing parent during pregnancy have been shown to be associated with obesity in the offspring,^35^ and gestational diabetes may further compound these effects.

Our results are consistent with prior studies that identified individual associations between either in-utero exposure to prenatal depression or gestational diabetes and childhood obesity. Evidence on prenatal depression supports an association with childhood adiposity.^39,40^ Systematic reviews focusing on the perinatal period report a positive association between birthing parent’s depression and childhood overweight or obesity, with greater associations observed among those experiencing longer depression durations.^11,40,41^ A US-based cohort study found that prenatal depression was associated with greater central adiposity in children.^42^ Notably, many prior studies rely on birthing parent self-reports of depressive symptoms rather than clinical diagnoses. In contrast, our study leveraged clinical diagnoses and symptom severity captured by a validated instrument.

In-utero exposure to gestational diabetes has been associated with childhood obesity.^43,44^ A recent meta-analysis of over 160 000 birthing parent–child pairs found an increased odds of childhood overweight or obesity (odds ratios ranging between 1.32 and 1.59 depending on life stage) among children born to individuals with gestational diabetes compared with individuals without gestational diabetes.^4^ Similar to our study, the meta-analysis documented a slightly attenuated, but statistically significant association with childhood obesity up to age 10 years after adjustment for prepregnancy BMI.^4^

### Strengths and Limitations

Study strengths include a large, racially and ethnically diverse cohort, enhancing generalizability. Standardized, validated protocols and instruments reduced potential exposure and outcome misclassification, including universal gestational diabetes screening, with the majority of the gestational diabetes cases diagnosed on the basis of objective plasma glucose values, which many prior studies lacked. Use of EHR data minimized recall bias and enabled longitudinal follow-up of childhood obesity risk through age 10 years. Although the oldest age group (44 894 children) was smaller than the youngest (199 329 children), it was still sizable, although some censoring bias remains possible. Across the 3 life-stage analyses, covariate demographics were comparable, reducing attrition concerns.

Regarding limitations, causal inference cannot be established in an observational study, and residual confounding may persist despite adjustment for key covariates identified in prior literature. Postnatal factors (eg, diet and physical activity, which can influence childhood BMI), were not included, as the study focused on prenatal exposures. Although prenatal depression was clinically diagnosed using standardized protocols, some underreporting of depressive symptoms through the PHQ-9 may have occurred, which could lead to association underestimation. Additionally, we included only singleton pregnancies and selected the eldest child per birthing parent to ensure independence and avoid bias from correlated outcomes or later fertility decisions, although this approach may have slightly underestimated gestational diabetes prevalence and yielded conservative effect estimates. Despite these limitations, our targeted focus on prenatal risk factors within a large, integrated health system provides valuable insight into early determinants of childhood obesity.

## Conclusions

In this cohort study, both prenatal depression and gestational diabetes were associated with obesity risk in the offspring, with gestational diabetes exhibiting larger effect sizes with greater clinical relevance. Children exposed to both conditions had highest relative risks, particularly at ages 5.0 to 7.9 years, although associations appeared additive rather than synergistic. These results highlight the importance of universal prenatal screening and risk stratification with targeted interventions for children who were exposed in utero to these conditions.

## Notes

Supplement 1. eAppendix. Supplemental Methods eFigure 1. Study Consort Diagram eTable 1. Comparison of participant characteristics for those excluded from the analytic sample eFigure 2. Child BMI and BMI Z-Scores Longitudinally from 2-10 Years eTable 2. Association Between Gestational Diabetes Exposure and Childhood Obesity Risk (≥95th Percentile) Across Age Groups by Prenatal Depression Status and PHQ-9 Severity

Supplement 2. Data Sharing Statement
