# Supplementary material for: Joint and Independent Associations of Gestational Diabetes and Depression With Childhood Obesity
Source: JAMA Netw Open. 2026 Feb 18;9(2):e2559344. doi: 10.1001/jamanetworkopen.2025.59344 (PMC12917676; doi:10.1001/jamanetworkopen.2025.59344)
Supplement: Supplement 1. — eAppendix. Supplemental Methods eFigure 1. Study Consort Diagram eTable 1. Comparison of participant characteristics for those excluded from the analytic sample eFigure 2. Child BMI and BMI Z-Scores Longitudinally from 2-10 Years eTable 2. Association Between Gestational Diabetes Exposure and Childhood Obesity Risk (≥95th Percentile) Across Age Groups by Prenatal Depression Status and PHQ-9 Severity [file jamanetwopen-e2559344-s001.pdf]

Supplemental Online Content

Peterson AK, Avalos LA, Zhu Y, et al. Joint and independent associations of gestational diabetes and depression with childhood obesity. *JAMA Netw Open*. 2026;9(2):e2559344. doi:10.1001/jamanetworkopen.2025.59344

eAppendix. Supplemental Methods

eFigure 1. Study Consort Diagram

eTable 1. Comparison of participant characteristics for those excluded from the analytic sample

eFigure 2. Child BMI and BMI Z-Scores Longitudinally from 2-10 Years

eTable 2. Association Between Gestational Diabetes Exposure and Childhood Obesity Risk (≥95th Percentile) Across Age Groups by Prenatal Depression Status and PHQ-9 Severity

This supplemental material has been provided by the authors to give readers additional information about their work.

© 2026 Peterson AK et al. *JAMA Network Open*.

eAppendix. Supplemental Methods:

Prenatal depression was defined using, ICD-9 codes (296.2, 296.21, 296.22, 296.23, 296.24, 296.25, 296.3, 296.31, 296.32, 296.33, 296.34, 296.35, 298, 300.4, 309, 309.1, 311, 648.4), and ICD-10 codes (F32.0, F32.1, F32.2, F32.3, F32.4, F32.9, F33.0, F33.1, F33.2, F33.3, F33.41, F33.9, F34.1, F43.21, O99.34) available within the EHR.

Antidepressant medications included SSRIs (citalopram, escitalopram, fluoxetine, fluvoxamine, paroxetine, sertraline), tricyclic antidepressants (amitriptyline, clomipramine, desipramine, nortriptyline, doxepin, imipramine, protriptyline, trimipramine), SNRIs (desvenlafaxine, duloxetine, milnacipran, venlafaxine), monoamine oxidase inhibitors (phenelzine, tranylcypromine), and other medications (trazodone, bupropion, atomoxetine, mirtazapine, nefazodone, vilazodone).

© 2026 Peterson AK et al. *JAMA Network Open*.

eFigure 1. Study Consort Diagram

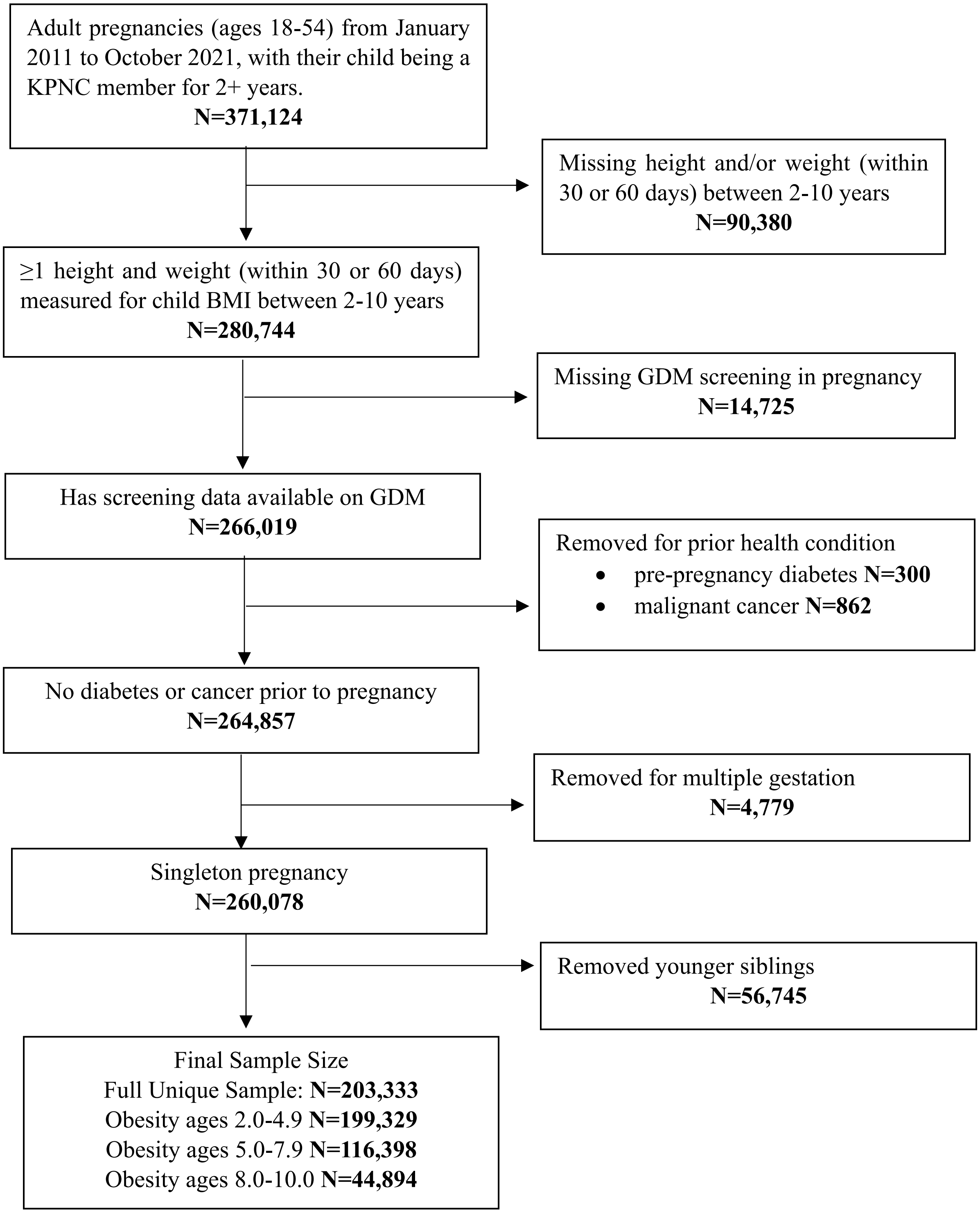

© 2026 Peterson AK et al. *JAMA Network Open*.

eTable 1. Comparison of participant characteristics for those excluded from the analytic sample

	Among first pregnancies during study period (N=284540)		Among first pregnancies during study period and singleton pregnancies (N=279403)	
	Multiple gestation	Singleton	Missing infant BMI or missing GDM screening	Has data on infant BMI and GDM screening
	(N=5137)	(N=279403)	(N=78165)	(N=201238)
**Maternal age (Mean ± SD)**	32.5 ± 5.6	30.5 ± 5.4	29.8 ± 5.7	30.8 ± 5.3
**Pre-pregnancy BMI (Mean ± SD)**	26.9 ± 6.5	26.5 ± 6.2	26.8 ± 6.6	26.3 ± 6.1
**Gestational age, weeks (Mean ± SD)**	35.8 ± 2.9	39.2 ± 1.8	39.1 ± 2.2	39.3 ± 1.7
**Maternal age**				
1. <25	407 (7.9)	40741 (14.6)	15318 (19.6)	25423 (12.6)
2. 25-29	1029 (20.0)	74270 (26.6)	21339 (27.3)	52931 (26.3)
3. 30-34	1889 (36.8)	98277 (35.2)	24736 (31.6)	73541 (36.5)
4. 35-54	1812 (35.3)	66115 (23.7)	16772 (21.5)	49343 (24.5)
**Race/ethnicity**				
Asian/Pacific Islander	1209 (23.5)	72704 (26.0)	17330 (22.2)	55374 (27.5)
Black	396 (7.7)	18813 (6.7)	6024 (7.7)	12789 (6.4)
Hispanic	1075 (20.9)	73682 (26.4)	22029 (28.2)	51653 (25.7)
Multiracial/Other	172 (3.3)	8033 (2.9)	1965 (2.5)	6068 (3.0)
Native American	22 (0.4)	1072 (0.4)	351 (0.4)	721 (0.4)
White	2180 (42.4)	100901 (36.1)	28910 (37.0)	71991 (35.8)
Unknown/Missing	83 (1.6)	4198 (1.5)	1556 (2.0)	2642 (1.3)
**Pre-pregnancy BMI**				
1. Underweight	350 (6.8)	29930 (10.7)	13470 (17.2)	16460 (8.2)
2. Normal	1884 (36.7)	103213 (36.9)	25304 (32.4)	77909 (38.7)
3. Overweight	1468 (28.6)	72522 (26.0)	17647 (22.6)	54875 (27.3)
4. Obese	1285 (25.0)	63127 (22.6)	17305 (22.1)	45822 (22.8)
Missing	150 (2.9)	10611 (3.8)	4439 (5.7)	6172 (3.1)
**Parity**				
1. 0	3098 (60.3)	156751 (56.1)	43468 (55.6)	113283 (56.3)
2. 1	1193 (23.2)	75787 (27.1)	20419 (26.1)	55368 (27.5)
3. 2+	838 (16.3)	46704 (16.7)	14178 (18.1)	32526 (16.2)
Missing	8 (0.2)	161 (0.1)	100 (0.1)	61 (0.0)
**Smoking, during pregnancy**				
No	5023 (97.8)	271164 (97.1)	74715 (95.6)	196449 (97.6)
Yes	112 (2.2)	7605 (2.7)	2843 (3.6)	4762 (2.4)
Missing	2 (0.0)	634 (0.2)	607 (0.8)	27 (0.0)
**Alcohol, during pregnnacy**				
No	4714 (91.8)	246018 (88.1)	67941 (86.9)	178077 (88.5)
Yes	396 (7.7)	30670 (11.0)	8078 (10.3)	22592 (11.2)
Missing	27 (0.5)	2715 (1.0)	2146 (2.7)	569 (0.3)
**Medicaid/Medicare**				
No	4593 (89.4)	248727 (89.0)	69846 (89.4)	178881 (88.9)
Yes	544 (10.6)	30676 (11.0)	8319 (10.6)	22357 (11.1)
**NDI**				
Q1	937 (18.2)	44622 (16.0)	11929 (15.3)	32693 (16.2)
Q2	1663 (32.4)	83249 (29.8)	21391 (27.4)	61858 (30.7)
Q3	1456 (28.3)	81187 (29.1)	22151 (28.3)	59036 (29.3)
Q4	1079 (21.0)	70090 (25.1)	22559 (28.9)	47531 (23.6)
Missing	2 (0.0)	255 (0.1)	135 (0.2)	120 (0.1)

© 2026 Peterson AK et al. *JAMA Network Open*.

eFigure 2. Child BMI and BMI Z-Scores Longitudinally from 2-10 Years

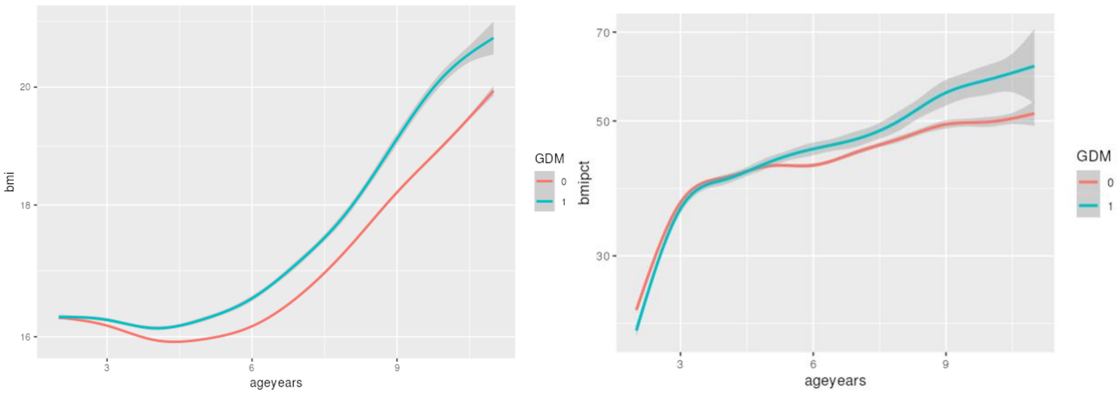

© 2026 Peterson AK et al. *JAMA Network Open*.

eTable 2. Association Between Gestational Diabetes Exposure and Childhood Obesity Risk (≥95th Percentile) Across Age Groups by Prenatal Depression Status and PHQ-9 Severity

		Stratified by Prenatal Depression					Stratified by PHQ-9 Score								
**Age**		**Prenatal Depression**		**No Prenatal Depression**		**P-for-interaction**	**0-4**		**5-9**		**10-14**		**15+**		**P-for-interaction**
	**Main Model**	**RR (95% CI)**	**Sample Size**	**RR (95% CI)**	**Sample Size**		**RR (95% CI)**	**Sample Size**	**RR (95% CI)**	**Sample Size**	**RR (95% CI)**	**Sample Size**	**RR (95% CI)**	**Sample Size**	
**2.0 – 4.9**	No GDM	Reference	17652	Reference	90541	0.35	Reference	66953	Reference	28636	Reference	8498	Reference	4106	0.48
	GDM	1.29 (1.16, 1.43)	1753	1.28 (1.23, 1.35)	9857		1.25 (1.18, 1.32)	6976	1.35 (1.25, 1.46)	3264	1.29 (1.12, 1.49)	934	1.30 (1.09, 1.57)	436	
	**Main Model + ppBMI**														
	No GDM	Reference	17652	Reference	90541	0.26	Reference	66953	Reference	28636	Reference	8498	Reference	4106	0.3
	GDM	1.07 (0.97, 1.19)	1753	1.08 (1.03, 1.14)	9857		1.04 (0.99, 1.11)	6976	1.14 (1.05, 1.23)	3264	1.12 (0.97, 1.30)	934	1.14 (0.95, 1.36)	436	
**5.0 – 7.9**	**Main Model**														
	No GDM	Reference	7109	Reference	39458	0.75	Reference	28842	Reference	12324	Reference	3629	Reference	1772	0.84
	GDM	1.45 (1.26, 1.67)	653	1.44 (1.36, 1.53)	4203		1.44 (1.33, 1.55)	2955	1.48 (1.34, 1.64)	1349	1.43 (1.18, 1.73)	374	1.27 (0.97, 1.66)	178	
	**Main Model + ppBMI**														
	No GDM	Reference	7109	Reference	39458	0.61	Reference	28842	Reference	12324	Reference	3629	Reference	1772	0.82
	GDM	1.15 (1.00, 1.33)	653	1.17 (1.11, 1.25)	4203		1.14 (1.06, 1.23)	2955	1.22 (1.10, 1.34)	1349	1.22 (1.01, 1.48)	374	1.07 (0.82, 1.40)	178	
**8.0 – 10.0**	**Main Model**														
	No GDM	Reference	474	Reference	2636	0.78	Reference	1922	Reference	813 (23.7)	Reference	256	Reference	119	0.15
	GDM	1.35 (0.75, 2.42)	41	1.49 (1.19, 1.86)	273		1.36 (1.02, 1.82)	192	1.94 (1.41, 2.68)	83 (2.4)	1.36 (0.45, 4.08)	23	0.56 (0.19, 1.71)	16	
	**Main Model + ppBMI**														
	No GDM	Reference	474	Reference	2636	0.38	Reference	1922	Reference	813 (23.7)	Reference	256	Reference	119	0.14
	GDM	1.08 (0.63, 1.87)	41	1.11 (0.89, 1.39)	273		0.98 (0.73, 1.30)	192	1.45 (1.07, 1.98)	83 (2.4)	1.39 (0.47, 4.16)	23	did not converge	16	

Main Model is adjusted for maternal age at time of delivery, race/ethnicity, NDI, parity, alcohol use in pregnancy, smoking in pregnancy
Main Model + ppBMI is Main Model additionally adjusted for pre-pregnancy BMI
Obesity is defined by a sex-and-age specific BMI z score ≥95th percent

© 2026 Peterson AK et al. *JAMA Network Open*.
