# Supplementary material for: Joint and Independent Associations of Gestational Diabetes and Depression With Childhood Obesity
Source: JAMA Netw Open. 2026 Feb 18;9(2):e2559344. doi: 10.1001/jamanetworkopen.2025.59344 (PMC12917676; doi:10.1001/jamanetworkopen.2025.59344)
Supplement: Supplement 2. — Data Sharing Statement [file jamanetwopen-e2559344-s002.pdf]

Data Sharing Statement

Peterson. Joint and Independent Associations of Gestational Diabetes and Depression With Childhood Obesity. *JAMA Netw Open*. Published February 18, 2026.
doi:10.1001/jamanetworkopen.2025.59344

Data
Data available: Yes
Data types: Deidentified participant data
How to access data: Please send data requests to alicia.k.peterson@kp.org and assiamira.ferrara@kp.org.
When available: With publication

Supporting Documents
Document types: None

Additional Information
Who can access the data: Researchers whose proposed use of the data has been approved.
Types of analyses: For a specified purpose.
Mechanisms of data availability: After approval of a proposal and with a signed data access agreement
